# Bioactive Lipophilic Antioxidants (Carotenoids, Tocols, Retinol, and Coenzyme Q_10_) in Human and Animal Tissues: Development and Validation of a Rapid Extraction and Chromatographic Method for Nutrition and Health Studies

**DOI:** 10.3390/antiox15010043

**Published:** 2025-12-29

**Authors:** Ana M. Benítez-González, Carla M. Stinco, Mladen Brnčić, Francisco J. Barba, Antonio J. Meléndez-Martínez

**Affiliations:** 1 Food Colour and Quality Laboratory, Facultad de Farmacia, Universidad de Sevilla, 41012 Sevilla, Spain; abenitez@us.es (A.M.B.-G.); ajmelendez@us.es (A.J.M.-M.); 2 Faculty of Food Technology and Biotechnology, University of Zagreb, Pierottijeva 6, 10000 Zagreb, Croatia; 3 Research Group in Innovative Technologies for Sustainable Food (ALISOST), Department of Preventive Medicine and Public Health, Food Science, Toxicology and Forensic Medicine, Faculty of Pharmacy and Food Sciences, Universitat de València, Avda, Vicent Andrés Estellés, 22, 46100 Burjassot, Valencia, Spain; francisco.barba@uv.es

**Keywords:** phytoene, phytofluene, tocopherols, tocotrienols, ubiquinone, vitamin A, vitamin E, antioxidants status

## Abstract

A rapid and robust analytical method was validated for the simultaneous extraction and quantification of carotenoids and other lipophilic antioxidants (tocopherols, tocotrienols, retinol and coenzyme Q10) in human and animal tissues using a tandem RRLC-DAD-FLD system. Thirty-eight compounds were identified, with limits of quantification as low as 0.001 µg for astaxanthin, retinol, and coenzyme Q10. Most analytes exhibited high recoveries (85–94%) and good precision (coefficient of variation < 10%), except for Co-Q10, which showed moderate variability. The method was applied to seven human tissue types and their corresponding animal tissues, demonstrating high versatility and analytical reliability. Several isomers of colourless carotenoids were identified in human tissues for the first time, reinforcing their emerging relevance in photoprotection and health. This method provides a valuable analytical tool for investigating the tissue distribution, bioavailability, functionality and nutritional significance of lipophilic antioxidants, thereby supporting future research on antioxidant status and health-related actions.

## 1. Introduction

Although numerous studies have investigated the role of bioactive compounds such as carotenoids and other lipophilic antioxidants (retinol, tocopherols, tocotrienols and coenzyme Q10) in reducing the risk of chronic degenerative diseases, the specific mechanisms through which they exert their protective effects remain incompletely understood. In this context, a deeper understanding of their tissue-specific distribution in the human body is essential, as it may yield critical insights into their biological roles and mechanisms of action.

Carotenoids are natural colourant compounds that are abundant in nature; with few known exceptions, animals cannot synthesise them de novo, so they must be ingested through the diet. Some carotenoids are provitamin A precursors and are versatile compounds for health promotion. They have many applications in agri-food, nutrition, health and cosmetics [1]. There is ample evidence that they are bioactives that contribute to reducing the risk of chronic diseases such as cancer, cardiovascular disease, neurological and metabolic disorders, among others [2]. They are also important for skin health (for instance, providing photoprotection against photooxidative damage) and appearance [2].

Retinol, an active form of vitamin A, is a fat-soluble micronutrient essential for several critical biological functions in humans. It plays a key role in vision, particularly in the regeneration of rhodopsin necessary for low-light (scotopic) vision. Additionally, retinol contributes to cell growth and differentiation, supports the maintenance of healthy skin and mucous membranes, and is vital for immune function and reproductive health [3,4,5].

Vitamin E comprises a group of fat-soluble compounds, primarily tocopherols and tocotrienols, which are collectively known as tocols. They have been associated with important health benefits, such as protection against certain types of cancer, neurodegenerative diseases, and eye disorders [6,7]. Among them, α-tocopherol is the most biologically active form and is widely recognised for its antioxidant properties, particularly by protecting cell membranes from lipid peroxidation and preserving the integrity of polyunsaturated fatty acids [8]. γ-Tocopherol, although less studied, exhibits distinctive antioxidant functions, particularly its ability to scavenge reactive nitrogen and oxygen species, suggesting a complementary role in human health [9].

Coenzyme Q10 (Co-Q10), also known as ubiquinone, is a lipid-soluble molecule found in all cellular membranes, especially within mitochondria. It is crucial for cellular energy production, facilitating electron transfer in the mitochondrial respiratory chain to generate ATP. Co-Q10 also serves as a powerful antioxidant, protecting cells from oxidative damage, preserving membrane integrity, and supporting mitochondrial function, particularly in high-energy-demand tissues like the heart [10,11].

All these health-promoting properties make the study of the accumulation of carotenoids, retinol, tocopherols and Co-Q10 in the different tissues of the human body a subject of interest. However, there is not much literature on the presence of these compounds in human tissues because of the difficulty in accessing healthy human tissue samples of different origins. The acquisition of such tissue is a complex and strictly regulated process that necessitates informed consent, full adherence to ethical and legal frameworks, and meticulous logistical procedures to ensure sample integrity.

Carotenoids are known to be present in human tissues and fluids, typically in the range of 0–2 μmol/L (plasma) and 0–1 nmol/g (tissues) [2]. The colourless carotenoids phytoene and phytofluene have been detected in plasma and tissues (adrenals, brain, lung, breast, liver, prostate, cervix, colon and skin) of animals at concentrations comparable to those of the other major dietary carotenoids [2,12,13]. Strikingly, there is very scarce information about levels of carotenoids in human tissues [14].

Table 1 summarises different published methods for the extraction and identification of carotenoids and other lipophilic antioxidants in human tissues. The main tissues studied were liver, kidney, lung, adrenal, testis, ovary, brain, cervix, prostate, breast and fat.

In most of the reviewed studies, the authors were able to identify α- and β-carotene, lutein, zeaxanthin and lycopene in all tissues. In the articles published before 2000, only the 9Z and 13Z isomers of β-carotene were identified, with the exception of Stahl et al. [16], who identified three Z-isomers of lycopene (9Z, 13Z and 5Z) and the 3- and 15Z isomers of β-carotene.

Since 2000, publications have identified not only carotenoids and Z-isomers, but also tocopherols, especially α-, δ- and γ-tocopherols. Particularly noteworthy is the work published by Harari et al. (2020) [27], in which they also identified ζ-carotene and the colourless carotenoids phytoene and phytofluene in human adipose tissue.

Retinol and Co-Q10 have been reported in mouse tissues [29,30] or in human serum [31,32], although the authors have only found one article where retinol is analysed in human tissues [26]. In that study, authors quantified retinol and other retinoids in human liver samples using a validated liquid chromatography–tandem mass spectrometry (LC–MS/MS) method with isotope-labelled internal standards. The retinol and retinyl palmitate contents were extracted from 40 to 60 mg of liver tissue through protein precipitation with cold acetonitrile, and analysed by LC–MS/MS under multiple reaction monitoring (MRM) mode. This approach allowed accurate and specific quantification of endogenous retinoids, even in the presence of complex matrix effects typical of liver tissue.

Similarly, only one study has been found in which Co-Q10 was quantified directly in human tissues. Montero et al. (2008) [28] analysed Co-Q10 concentrations in skeletal muscle and skin fibroblasts from patients with suspected mitochondrial disorders. The authors used a validated high-performance liquid chromatography method with electrochemical detection (HPLC-ECD) after extraction with hexane from muscle homogenates. The method enabled reliable quantification of total Co-Q10, and results were expressed both relative to total protein and to citrate synthase activity, allowing improved diagnostic accuracy even in patients with atypical biochemical profiles. This study highlights the value of direct tissue analysis for the identification of Co-Q10 deficiency syndromes.

The objective of this work was to develop and validate an efficient extraction method, followed by Rapid Resolution Liquid Chromatography (RRLC), for the identification and quantification of a broad spectrum of lipophilic bioactive compounds in human and animal tissues, including carotenoids (coloured and colourless, the latter largely ignored), tocols, retinol and Co-Q10, including in some cases Z and all-E isomers or derivatives. The method allows for the simultaneous detection of compounds with absorption in the UV–vis region and those with fluorescent emission, thanks to the use of two tandem-coupled detectors: a diode array detector (DAD) and a fluorescence detector (FLD). The high sensitivity of the FLD allows for the accurate quantification of tocols even at very low concentrations.

This analytical strategy represents a significant methodological advance, not only due to its capacity to simultaneously detect structurally diverse compounds, but also for its rapidity and applicability to complex biological matrices. Given the inherent difficulties in obtaining human samples, the information available on validated methods for the extraction, identification, and quantification of these compounds in human tissues is limited. Moreover, many existing protocols could be updated to reflect recent analytical advances, such as improved instrumentation, greener solvent use and modern chromatographic materials. The methodology developed and validated in this study offers a valuable tool for investigating the tissue distribution and potential physiological functions of these compounds in the human body, thus contributing to a deeper understanding of their relevance to health.

## 2. Materials and Methods

### 2.1. Chemicals and Standards

All solvents (methanol, methyl-tert-butyl ether, hexane, diethyl ether) were HPLC-grade and, together with ascorbic acid, were purchased from Merck (Darmstadt, Germany). PBS 10×sterile solution was acquired in canvax (Boecillo, Valladolid, Spain). Purified water was used in analyses from NANOpure^®^ DIamondTM (Barnstead International, Dubuque, IA, USA). Lutein, zeaxanthin, violaxanthin, zeinoxanthin, ζ-carotene, α-carotene, β-carotene, β-cryptoxantin, lycopene, phytoene, retinol, astaxanthin, capsanthin, neoxanthin, echinenone and Co-Q10 were from Sigma-Aldrich (St. Louis, MO, USA). Phytofluene was acquired from Carotenature GmbH (Münsingen, Switzerland). The tocopherols standards were from Calbiochem (Merck, Darmstadt, Germany), and tocotrienols were acquired from Extrasynthese (Lyon, France).

### 2.2. Samples and Experimental Design

The study of the antioxidant levels in human samples was approved by the ethical research committees of the “Hospitales Universitarios Virgen Macarena-Virgen del Rocío (Seville, Spain) (code 0654-N-18)”. A human liver sample provided by the Nodo of the HUVR Biobank, which has all the necessary ethical permits to obtain human tissue samples from healthy patients, was used to validate the extraction procedure and determine its precision and accuracy. This sample was used for two reasons: it was possible to obtain an adequate sample size to perform all tests on the same sample, resulting in a more robust model. Secondly, the liver is an organ that has been shown to have a very diverse carotenoid profile.

Seven different human tissues and the livers of two animal species (cow and chicken) (obtained from a local butcher’s shop) were selected as samples to evaluate the applicability of the method under validation, as they were expected to exhibit a broad profile of carotenoids, tocopherols, Co-Q10 and retinol.

### 2.3. Extraction of Lipophilic Antioxidants

Approximately 0.05–0.10 g of sample was weighed in a 2 mL Eppendorf tube, and 1 mL of a solution of PBS + ascorbic acid 2.5% was added. A milling step was applied with a ball mill (MM 400, Retsch, Haan, Germany) for 5 min with a frequency of 30 Hz. The stainless-steel balls were stored in the freezer to prevent heating during the process.

The homogenate was transferred to a 50 mL tube, and the Eppendorf tube was washed with PBS + ascorbic acid 2.5% to remove all the sample. The final volume was 2 mL approximately. Next, 2 mL of absolute ethanol and 4 mL of 20% methanolic potassium solution were added. Samples were kept 30 min under gentle agitation, N_2_ atmosphere (to avoid oxidation) and at room temperature.

After this time, 8 mL of 10% NaCl and 5 mL of hexane were added, then the samples were shaken in a Vortex for 5 min at 2500 rpm and centrifuged in an Eppendorf Centrifuge 5810R (Brinkman Instruments Inc., Westbury, NY, USA) for 5 min at 4 °C and 3900× *g*.

After recovering the coloured fraction, the residue was extracted again with another aliquot of 5 mL of hexane. The two extracted fractions were pooled and concentrated to dryness in a rotary evaporator under vacuum (Concentrator Plus, Eppendorf, Germany) and stored under N2 atmosphere until RRLC analysis.

Before the injection, the dried extracts were dissolved in ethyl acetate and centrifuged at 18,000× *g*, 4 °C for 5 min.

### 2.4. RRLC Analysis

The chromatographic method used was the one validated by Stinco et al. (2019) [33] with some modifications. The system used was an Agilent 1260 system equipped with a DAD which was set to 285 nm for the colourless carotenoid phytoene, Co-Q10 and for tocopherols, 325 nm for retinol, 350 nm for the colourless carotenoid phytofluene, 410 nm for ζ-carotene, 472 nm for lycopene and 450 nm for the rest of the carotenoids. Furthermore, the equipment was equipped with a fluorescence detector (FLD), which was selected at 295 nm in excitation and 325 nm for emission in order to detect tocotrienols. The column was a YMC (YMC Europe, Germany) C30 column (150 × 4.6 mm, 3 µm particle size) preceded by a YMC pre-column. The mobile phase consisted of Methanol (A), methyl-tert-butyl ether (B) and water (C) at 1 mL/min flow.

The identification of the compounds was performed by comparing their retention times and UV/vis spectroscopic characteristics with those of the commercial standards. External calibration was used for quantification. The identification of *Z*-isomers in the human tissues was carried out by comparing their chromatographic and spectroscopic profiles with data reported in the literature [34,35,36]. All isomers were quantified using the calibration curve constructed with the corresponding all-*E* standard.

### 2.5. Method Validation

The method was validated according to internationally recognised guidelines UNE-82009-2 [37], assessing its homocedasticity, linearity, precision (repeatability, reproducibility), accuracy, and sensitivity (limit of detection (LOD) and limit of quantification (LOQ)).

#### 2.5.1. Calibration Curves, Linearity and LOD and LOQ

The linearity of the method was assessed by analysing the detector response (area units) to various amounts (g) of lipophilic antioxidants using linear regression. Homoscedasticity and linearity were verified through the F-test and residual plot analysis (95% significance level) [38].

To quantify the concentration of the standards, a UV–vis spectrophotometer Agilent 8453 (Agilent Technologies, Santa Clara, CA, USA) was used, and the corresponding molar absorptivity values were considered [39]. Standard curves were generated by plotting the response of various dilutions of the quantified standards against the injected concentration.

The LOD and LOQ were calculated from the calibration curves as three and ten times the relative standard deviation of the analytical blank, respectively.

#### 2.5.2. Accuracy and Precision of the Method

The accuracy of the method was determined by recovery studies using the standard addition method, for which echinenone, a-tocopherol, retinol and coenzyme Q10 (Co-Q10) were selected as standards. For precision (repeatability and reproducibility), a non-spiked human liver sample was used, as it naturally contained quantifiable levels of the studied analytes. For accuracy (recovery), the same liver sample was spiked at different levels, and the endogenous content was subtracted from the final calculation.

The recovery study was performed by spiking a human liver sample with these standards and then extracting as described above (Section 2.3), and subsequently the spiked samples were analysed with the proposed RRLC method. To ensure the method’s applicability in a wide range of biological matrices, recovery experiments were performed at different concentration levels. Specifically, two fortification levels (low and high) were used for compounds detected by DAD and three levels (high, medium and low) for those detected by FLD, to ensure a wide concentration variability. This strategy allowed for method validation under realistic conditions and across a concentration spectrum relevant to different tissue types.

The assay was performed in triplicate. The recovery was calculated by comparing the values obtained for each standard with the initial value added to the sample.

The precision of the method was assessed by evaluating repeatability (intra-day) and reproducibility (inter-day). Repeatability was determined by analysing the content of the studied compounds in three replicates of the same sample under consistent analytical conditions. Reproducibility was evaluated by extracting and analysing the same sample at two-day intervals over a period of three days. Precision was expressed as the relative standard deviation (RSD%).

## 3. Results and Discussion

### 3.1. Determination of Conditions

In order to determine the extraction conditions, a bibliographic review of existing tissue extraction methods was conducted. As summarised in Table 1, thirteen methods were identified, more than 70% of which were published before 2004, i.e., more than 20 years ago. In light of current knowledge, certain steps and conditions are no longer recommended, such as the application of high temperatures or the use of specific reagents, as these can lead to losses (oxidation/degradation) or structural modifications (isomerisation) of the compounds to be extracted.

From the extraction methods available in the literature, the following conditions were compared: sample preparation and homogenization, addition of antioxidants, extraction solvents and saponification conditions. In addition, tests were conducted to establish the optimal conditions for identifying the compounds of interest.

#### 3.1.1. Sample Preparation and Homogenisation

It is well known that sample homogenisation is a critical step in the extraction of lipophilic compounds, especially when dealing with a heterogeneous matrix such as tissues. In the proposed method, a grinding step of the samples was performed in a micro-ball mill; this step allowed a better extraction of the sample, as a completely homogenised sample was obtained. This step was not found in the published methods (Table 1), which instead used grinding in a mortar with liquid nitrogen, sonication or did not perform this step at all.

#### 3.1.2. Addition of Antioxidants

Several reported methodologies used BHT as an antioxidant. Tests were performed using 0.1% BHT in ethanol to ensure that the antioxidant did not cause chromatographic interference with phytoene and phytofluene. It was found that there were no interferences, but that the use of BHT caused a peak in the chromatogram which made interpretation of the other peaks difficult. Its use was therefore discarded.

#### 3.1.3. Extraction Solvents

An extraction test was performed with ethyl ether to extract carotenoids. We found that the use of this solvent created an interface, which meant that the extract did not reach dryness. Hexane was chosen because, as shown in Table 1, most authors use this solvent for the extraction. Being a non-polar solvent, it allowed not only a better separation of the phases, but also an improvement in the concentration of the extract.

#### 3.1.4. Saponification Conditions

As can be seen in Table 1, there is no consensus in the literature regarding the use of a saponification step or its optimal conditions. In studies that include one, the protocols vary significantly in terms of reagent, concentration, temperature and duration: Clinton et al. (1996) [18] used 1 mL of saturated KOH for 30 min at 70 °C; El-Sohemy et al. (2002) [21], KOH for 15 min at 50 °C; Vishwanathan et al. (2013) [25], 1 mL of 5% NaOH for 20 min at 60 °C; and Harari et al. (2020) [27], 12% KOH in ethanol for 30 min at 50 °C.

Given this methodological heterogeneity, we decided to apply the conditions typically used in our laboratory, previously validated by Stinco et al. (2014) [40], with slight modifications; 30% KOH in methanol was used for one hour, under gentle stirring, under a nitrogen atmosphere, protected from direct light, and without applying heat. This procedure aims to minimise the degradation of the target compounds and prevent the formation of artefacts, thus ensuring greater reproducibility and analytical specificity.

### 3.2. Linearity and Limits of Detection and Quantification

Table 2 summarises the validation parameters: slope, intercept, coefficients of determination, LOD and LOQ. The homoscedasticity of the linear calibration range was assessed to confirm the applicability of the linear least-squares method (constant variance).

The linearity coefficients within the selected concentration range were very high. In the case of compounds detected by DAD, they were R^2^ > 0.999 for most compounds, except for neoxanthin and zeaxanthin, which were 0.997 and 0.996, respectively.

For tocols detected by FLD, these coefficients were also excellent, greater than R^2^ > 0.999 for all except α-tocotrienol, which was 0.997.

The DAD detection limits ranged from 0.0004 μg for β-carotene to 0.054 μg for α-tocotrienol. Of particular note is the case of Co-Q10, whose LOD was 0.00004 μg. In the case of compounds detected by FLD, the detection limits ranged from 0.0001 µg for δ- and γ-tocopherol to 0.001 µg for the remaining compounds.

The LOQ varied between 0.0001 μg for Co-Q10 and 0.181 μg for α-tocotrienol for compounds detected on DAD and between 0.003 μg for α-tocopherol and 0.005 μg for α-tocotrienol when using the FLD detector.

These results confirm that this method is rapid and sensitive for the detection and quantification of carotenoids, tocopherols, retinol and Co-Q10.

### 3.3. Accuracy

The accuracy of the extraction method was evaluated by calculating the mean percentage recovery of spiked standards α-tocopherol, Co-Q10, retinol and echinenone for carotenoids in human liver samples. The standards were spiked at two concentrations, high and low, and in the case of tocopherols, at three concentrations (high, medium and low) to include recovery by fluorescence detection. Samples fortified with the standards were extracted using the developed method and analysed by RRLC. All assays were performed in triplicate. The amounts spiked and recovered, as well as the mean and standard deviation of the percentage recovery, are given in Table 3.

For both retinol and α-tocopherol, the recoveries were above 90% for both high and low added concentrations. In addition, for tocopherols, a recovery of 91.5% was obtained by fluorescence detection. For echinenone, recovery was 88.7% for high concentrations and 84.6% for low concentrations.

Particularly striking was the case of Co-Q10, of which only 38% was recovered. This low recovery percentage could be due to the instability of coenzyme Q10, particularly due to the saponification process. Turkowicz and Karpińska (2013) [41] published a review of all the analytical problems associated with the analysis of Co-Q10 in biological samples. Indeed, in matrices such as plasma, higher Co-Q10 recovery rates have been reported [42], likely due to the lower lipid content and simpler composition compared to our samples. However, in our specific case, the complexity and lipid richness of the matrix require saponification despite the associated risks of Co-Q10 degradation. However, despite the degradation problems associated with the saponification process, the recovery of Co-Q10 showed standard deviations of less than 3%, demonstrating the robustness and reproducibility of the method even under complex analytical conditions.

### 3.4. Precision

The precision of the method (expressed as repeatability and reproducibility) was evaluated by the relative standard deviation (RSD%); the values are tabulated in Table 4. The repeatability (intra-day) was less than 10% for practically all the compounds studied, except for lutein, for which the deviation was 10.19%. In terms of reproducibility, all the compounds, with the exception of Co-Q10, showed a RSD% of less than 10%. This could be mainly because this compound undergoes a change with the saponification process, in which several peaks of this compound can be seen chromatographically. Abdul-Rasheed and Farid (2009) [43] developed a method for the extraction of Co-Q10 from plasma that allows high recoveries; in our case, it is not possible to apply a one-step method without saponification, since human tissues usually contain fats, and the saponification process is key to the identification of carotenoids.

Boulet et al. (2020) [44] developed a method to determine retinol, six carotenoids, two tocopherols and Co-Q10 from human plasma by HPLC; their intra-assay precision (RSD%) results were also less than 10%. In this case, they also did not perform a saponification step in the sample, as this is not necessary in plasma.

In summary, considering the deviations recorded, the extraction method is a robust method with high repeatability and reproducibility.

### 3.5. Analysis of Carotenoids, Tocopherols and Coenzyme Q10 in Different Tissues

To validate the proposed method, carotenoids, tocopherols, retinol and coenzyme Q_10_ were extracted, identified and quantified in two animals (cow and chicken liver) and seven human tissue types.

A total of 38 different compounds were identified in the different tissues, including tocopherols, carotenoids, retinol and coenzyme Q (Table 5).

α-Tocopherol was detected in all samples analysed, with the highest concentrations found in human intestinal tissue and animal liver, with similar average values of approximately 4 µg/g of tissue. Notably, the concentration in the human intestine was eight times higher than in the pancreas, spleen, and liver (average concentrations of 0.50 µg/g), and approximately 60 times higher than in the duodenum (0.07 µg/g).

Among the colourless carotenoids, four *Z*-isomers of phytoene and four *Z*-isomers of phytofluene were identified. Phytoene was detected in all samples, except in human duodenum, whereas phytofluene was not detected in animal tissues. The tissues with the highest concentrations of colourless carotenoids were liver and intestine, with a mean value of 0.27 µg/g of tissue. Harari et al. (2020) [27] reported that phytoene accounts for approximately 8% of total carotenoids in human adipose tissue and phytofluene for 17%. In their analysis, one isomer of phytoene and four isomers of phytofluene were detected using HPLC. In our study, similar proportions were found in the intestine, with phytoene representing 3.2% and phytofluene 19.3% of the total carotenoid content. In the remaining samples, concentrations were highly variable, with colourless carotenoids accounting for between 2.4% and 12.7% of the total.

Among the macular carotenoids, we identified all-*E*-lutein and one *Z*-isomer, as well as all-*E*-zeaxanthin and three *Z*-isomers. In contrast, neither Harari et al. (2020) [27] nor Peng et al. (1993) [17] reported geometric isomers of zeaxanthin in their studies. Harari et al. identified only one *Z*-isomer of lutein in human adipose tissue and did not specify isomeric forms of zeaxanthin. They reported that zeaxanthin represented approximately 2% of total carotenoids in adipose tissue. In our study, the relative zeaxanthin content varied substantially, ranging from 0.76% in human liver to as high as 60% in cow and chicken livers. Regarding absolute concentrations, Peng et al. (1993) [17] reported 0.05 µg/g of lutein and 0.08 µg/g of zeaxanthin in human liver. Our results were consistent with these values, with 0.03 µg/g of lutein and 0.04 µg/g of zeaxanthin in the same tissue. Similarly, for the spleen, our concentrations of 0.05 µg/g lutein and 0.02 µg/g zeaxanthin closely matched those reported by Peng et al. (0.04 µg/g and 0.05 µg/g, respectively). Notably, carotenoid concentrations in animal livers were substantially higher than those observed in human liver. Specifically, lutein levels were approximately ten times greater, and zeaxanthin levels were about twice as high, suggesting a possible species-related difference in hepatic carotenoid accumulation.

β-cryptoxanthin was found in all human tissues analysed, but not in the animals. The concentration of β-cryptoxanthin in our samples ranged from 0.001 (pancreas) to 0.096 (liver) µg/g tissue, falling within the range reported in human tissues by previous studies. For instance, Schmitz et al. (1991) [15] reported values between 0.04 and 0.26 µg/g in liver, kidney and lung, while Clinton et al. (1996) [18] found 0.11 µg/g in prostate tissue. These comparisons support the validity of our measurements and highlight the expected variability across tissue types.

The provitamin A carotenoids α- and β-carotene were found in all human and animal samples, except in the pancreas, where α-carotene was not detected. In our study, we identified three *Z*-isomers of β-carotene in addition to all-*E*-β-carotene, and one *Z*-isomer of α-carotene along with the all-*E* form. These findings are in line with those of Harari et al. (2020) [27], who also detected multiple *Z*-isomers of both α- and β-carotene in human adipose tissue, specifically three for β-carotene and two for α-carotene. Similarly, Gamboa-Pinto et al. (1998) [19] and Peng et al. (1993) [17] reported the presence of *Z*-isomers of β-carotene in human cervical and solid tissues, although without fully quantifying their number. The β-carotene concentrations measured in our human samples ranged from 0.005 µg/g in pancreas to 0.541 µg/g in human liver, with intermediate levels in duodenum (0.009 µg/g), intestine (0.011 µg/g), spleen (0.050 µg/g), adipose tissue (0.008 µg/g) and human intestinal tissue (0.127 µg/g). In the liver, our value of 0.541 µg/g was substantially higher than the 0.145 µg/g reported by Schmitz et al. (1991) [15]. For adipose tissue, our result (0.008 µg/g) was lower than the values reported by Yeum et al. (1998) [20] and Chung et al. (2009) [24] (0.038–0.050 µg/g), which may reflect differences in anatomical site, fat content or population. β-carotene in the intestine and duodenum has been rarely quantified directly in prior work; thus, our data (0.009–0.127 µg/g) contribute novel values to the literature.

The α-carotene concentrations in our samples ranged from 0.001 µg/g in pancreas to 0.065 µg/g in human liver. In animal liver, levels were lower, with 0.004 µg/g in cow and 0.008 µg/g in chicken samples. When compared to literature values, our results are consistent with those reported by Stahl et al. (1992) [16], who found α-carotene concentrations in human liver ranging from 0.03 to 0.52 µg/g. Similarly, our adipose tissue value (0.004 µg/g) fell within the range described by the same authors (not detected to 0.15 µg/g). In contrast, Harari et al. (2020) [27] reported that α-carotene represented approximately 14% of total carotenoids in human adipose tissue, whereas in our case, this proportion was notably lower, at 2.5%.

Regarding lycopene, we identified the all-*E*-lycopene isomer along with five *Z*-isomers, including 15*Z*-, 13*Z*- and 5*Z*-lycopene, as well as two additional *Z*-isomers that could not be identified. Similarly, Clinton et al. (1996) [18] quantified lycopene in prostate tissue and identified all-E-lycopene together with 9*Z*-, 13*Z*- and 15*Z* isomers, confirming the presence of structurally distinct forms in human tissues.

The total lycopene concentration (sum of all isomers) in our study ranged from 0.02 µg/g in the pancreas to 2.77 µg/g in human liver. In contrast, lycopene was not detected in any of the animal liver samples. In adipose tissue the total lycopene concentration was 0.04 µg/g and 2.77 µg/g in the liver. These values are consistent with those reported by Stahl et al. (1992) [16], who quantified lycopene (without isomer distinction) in human liver within a range of 0.05–2.10 µg/g and from not detected to 0.27 µg/g in adipose tissue. Antwi et al. (2016) [45] also assessed adipose tissue, reporting a total lycopene concentration of 0.32 µg/g, combining *Z* and all-*E* isomers, although they did not specify individual forms. In contrast, Chung et al. (2009) [24] provided a more detailed isomeric analysis in freeze-dried adipose tissue, identifying all-*E*-lycopene along with 15*Z*-, 13*Z*-, 9*Z*- and 5*Z*- isomers, as well as one unidentified *Z* isomer. Their reported concentration was 1787.24 µg/g dry weight, markedly higher than values typically found in the literature, including our study, probably due to a large step to the lyophilisation step prior to extraction. Regarding relative abundance, Harari et al. (2020) [27] estimated that lycopene represents approximately 12% of total carotenoids in human adipose tissue, whereas in our samples, this proportion reached 34%, highlighting the high lycopene contribution in our adipose dataset.

In addition to the major carotenoids, we detected three isomers of ζ-carotene, as well as capsanthin and zeinoxanthin. Among the animal liver samples, zeinoxanthin was the only one consistently present, with concentrations of 0.076 µg/g in cow liver and 0.049 µg/g in chicken liver, values that closely matched those found in human liver (0.063 µg/g).

In our study, the total ζ-carotene concentration (sum of the three isomers) ranged from not detected in the pancreas to a maximum of 0.146 µg/g in the liver. In adipose tissue, the concentration was 0.004 µg/g. One of the few published references reporting ζ-carotene in human tissues is the study by Qin et al. (2008) [23], who detected two isomers in human breast adipose tissue, with a mean total concentration of 0.32 µg/g. Our data are among the few to report ζ-carotene levels across multiple human and animal tissues. The reported data about the levels of zeinoxanthin or capsanthin in human tissues are very scarce at best. While both carotenoids have been described in food matrices and some bioavailability studies, there is no scientific literature documenting their concentrations in specific human organs or adipose tissue. Therefore, the detection and quantification of these compounds in our samples may represent interesting findings, highlighting the originality and potential relevance of our study.

We also identified two isomers of Co-Q10, with concentrations ranging from not detected in spleen to 0.123 µg/g in human liver. In animal samples, Co-Q10 was not detected in bovine liver, whereas it was present in chicken liver at 0.314 µg/g, a value significantly higher than that observed in human tissue. These interspecies differences may be related to physiological and metabolic factors and dietary intake, all of which influence tissue accumulation. In line with our findings, Bhagavan and Chopra (2006) [46] reported that the liver is among the human organs with the highest Co-Q10 content, with concentrations reaching approximately 34.2 µg/g of tissue. The considerably lower concentrations observed in our study are likely due to a combination of methodological and biological factors. Firstly, the saponification step, essential for the analysis of carotenoids in fatty matrices, significantly degrades Co-Q10 (recovery ~38%, Table 3) compared to direct extraction methods. Secondly, inter-individual variability regarding diet, metabolic status and age can strongly influence tissue levels. Finally, the use of biobank samples, which may have experienced varying post-mortem intervals prior to freezing, could have contributed to the degradation of this labile compound compared to fresh tissue studies.

Retinol was identified in all analysed tissues except the duodenum, where it was not detected. Concentrations ranged from not detected to 6.202 µg/g in human liver. In animal samples, retinol levels were significantly higher than those observed in human tissues, with concentrations of 86.710 µg/g in cow liver and 80.085 µg/g in chicken liver. The concentrations detected in human liver are consistent with those reported by Zhong et al. (2019) [26], who found 4.04 µg of retinol per gram of liver tissue. It is well established that the liver is the primary storage site of vitamin A in the body. Between 66% and 75% of dietary retinoids (from chylomicrons and their remnants) are taken up by the liver, where they are stored mainly in hepatic stellate cells, while the remaining fraction is distributed to peripheral tissues (D’Ambrosio et al., 2011 [5]). Our results are fully in line with this physiological pattern, with the highest retinol concentrations observed in the liver.

Finally, some limitations of this study must be acknowledged. Firstly, the procurement of healthy human tissues is subject to strict ethical and logistical rules, which limited our sample size and prevented analysis of a larger population. Secondly, as discussed above, the saponification step required for the extraction of carotenoids from fatty matrices causes partial degradation of labile compounds such as Co-Q10, resulting in an underestimation of their absolute concentration compared to specific non-saponified methods. Despite these limitations, the method proved to be robust for the complete profile of the main lipophilic antioxidants in various tissue types.

## 4. Conclusions

A rapid, validated extraction method has been proposed to analyse carotenoids and other lipophilic antioxidants, such as tocols, retinol and Co-Q10, in human and animal tissues. A total of 38 compounds were identified across the analysed samples, including both Z-isomers and all-E-isomers. The applicability of the method was confirmed through validation criteria covering linearity, repeatability, reproducibility and accuracy. The method was successfully applied to seven different human tissues, as well as corresponding animal tissues for comparative purposes, thus establishing its robustness and versatility in investigating the distribution of these compounds in the body.

However, due to ethical and logistical limitations in accessing human tissue samples, research in this area remains scarce. Consequently, knowledge of organ-specific carotenoid distribution in humans is limited, hindering a full understanding of their potential roles in health promotion and disease prevention. Most recent studies have focused on the skin, either using indirect, non-invasive techniques or measuring plasma.

In contrast, our study provides novel data by identifying previously unreported carotenoids in human tissues and expanding the known carotenoid profiles of several organs. Of particular note is the detection of colourless carotenoids, which are increasingly recognised as being relevant to health and protection against UV radiation.

## Figures and Tables

**Table 1:** Literature review of published methods for the extraction and identification of carotenoids, tocopherols, retinol and Co-Q10 in human.

Matrix	Homogenisation/Pre-TREATMENT	Saponification/Digestion	Extraction Solvent	Analysis System	Compounds	Reference
Liver, kidney, lung	Homogenised in ethanol:water (50:50) w/1% BHT (45–90 s)	10% NaOH in ethanol, 60 °C, 30 min	Hexane (3 × 10 mL)	HPLC	α-carotene, β-carotene, cryptoxanthin, lutein, lycopene	[15]
Liver, kidney, adrenals, fat, testes, ovary, brainstem	Ground under liquid N_2_; Homogenised in buffer (pH 7.2) + SDS	None (SDS treatment)	2-Propanol (1 mL) + n-Hexane/Dichloromethane (5:1, 6 mL)	HPLC	all-*E*-lycopene, 9*Z*, 13*Z*, and 15*Z*-lycopene, 13*Z* and 15*Z*-β-carotene, all-*E*-β-carotene	[16]
Facial skin, cervical, ovarian	Enzymatic digestion: Collagenase (37 °C, 1 h) + Protease (37 °C, 30 min)	Enzymatic only (no alkaline saponification)	SDS-ethanol solution + Hexane (2 × 500 µL)	HPLC	Lutein, zeaxanthin, β-cryptoxanthin, lycopene, α-carotene, all-*E*-β-carotene, *Z*-β-carotene, retinol, α-tocopherol and β-tocopherol	[17]
Prostate	Minced and homogenised in ethanol w/0.1% BHT	Saturated KOH, 70 °C, 30 min	Hexane (3 × equal volumes)	HPLC	all-*E*-Lycopene,9*Z-*, 13*Z-*, 15*Z*-lycopene, all-*E*-β-carotene, 9*Z*-β-carotene, α-carotene, lutein, α-cryptoxanthin, zeaxanthin and β-cryptoxanthin	[18]
Cervical tissue	Enzymatic digestion: Collagenase + Ascorbic acid (37 °C, 1 h); Mechanical (<60 s)	Enzymatic only	Hexane w/BHT (3 × 2 mL)	HPLC	α-carotene and β-carotene	[19]
Breast adipose	Incubation w/12% pyrogallol in ethanol	30% KOH, 37 °C, 2 h	Ether/Hexane (2:1) (2 × 3 mL)	HPLC	Lutein, zeaxanthin, α- and β-cryptoxanthin, α-carotene, all-*E*-β-carotene, 13*Z*-β-carotene and lycopene. Retinol, all-*E* and 13*Z* retinoic acids, and α- and γ- tocopherol	[20]
Adipose tissue	Homogenised in 20% ascorbic acid solution	KOH, 50 °C, 15 min	Hexane	HPLC	α-carotene, β-carotene, β-cryptoxanthin, lycopene and lutein + zeaxanthin	[21]
Brain	Ground in mortar with Na_2_SO_4_	None	Hexane:Ethyl acetate (90:10)	HPLC	lutein, zeaxanthin, anhydrolutein, α-cryptoxanthin, ß-cryptoxanthin, α-carotene, *Z*- and (all-*E)*-ß-	[22]
Breast adipose	Incubation w/12% pyrogallol in ethanol	30% KOH, 37 °C, 2 h	Ether/Hexane (2:1) (3 mL + 2 mL)	HPLC	carotene, and *Z*- and all-*E*-lycopene and α-,γ-, δ-tocopherols	[23]
Adipose tissue	Lyophilised tissue	Incubation w/pyrogallol, 37 °C, 2 h	Ether/Hexane (2:1) (2 extractions)	HPLC	9*Z*-β-carotene, total β-carotene, ζ-carotene 1, ζ-carotene 2, total lycopene	[24]
Brain	Homogenised in saline/ethanol (0.3/0.5 mL)	5% NaOH + 25% Na-Ascorbate, 60 °C, 20 min	Hexane (2 × 5 mL)	HPLC	α-carotene, β-carotene (all-*E*, 15*Z*, 13*Z*, 9*Z*), lutein + zeaxanthin, lycopene (all-*E*, 15*Z*, 13*Z*, 9*Z*, 5*Z*,), β-cryptoxanthin	[25]
Liver	Mill homogeniser with ceramic beads in methanol-dry ice	None (Protein precipitation)	Acetonitrile (1 mL)	LC-MS/MS	Lutein and zeaxanthin	[26]
Adipose tissue	Extracted with ethanol w/BHT (10 μM)	12% KOH in ethanol, 50 °C, 30 min	Ethanol (2 mL) + Hexane (2 mL)	HPLC	α-carotene, β-carotene,ζ-carotene, lutein, lycopene, phytoene, phytofluene	[27]
Skeletal muscle	Mix with 1-propanol	None	n-hexane	HPLC-ECD	coenzyme Q10 (Total Co-Q10)	[28]

**Table 2:** Summary of data about linearity, LOD, LOQ, linear range and instrumental precision of the method.

Compounds		Wavelength		Equations	Correlation Coefficient		LOD	LOQ	Linear Range (µg)	
			(nm)		R^2^	SD_RES_	3xsD(A)/B	10xsD(A)/B	Min	Max
Carotenoids	α-carotene	DAD	450	y = 13,249.27x − 8.22	0.9999	7.19	0.001	0.003	0.008	0.165
	astaxanthin	DAD	472	y = 10,240.90x + 2.84	0.9999	7.92	0.001	0.003	0.001	0.169
	β-cryptoxanthin	DAD	450	y = 9174.29x + 5.14	1.0000	5.96	0.001	0.003	0.002	0.254
	β-carotene	DAD	450	y = 12,610.04x + 2.16	1.0000	3.93	0.0004	0.001	0.003	0.283
	capsanthin	DAD	450	y = 6131.24x − 15.19	0.9990	23.02	0.004	0.014	0.004	0.353
	lycopene	DAD	472	y = 15,598.81x − 36,74	0.9996	24.49	0.002	0.007	0.005	0.167
	lutein	DAD	450	y = 11,984.83x − 5.01	0.9999	10.37	0.001	0.003	0.002	0.179
	neoxanthin	DAD	450	y = 10,627.51x −26.18	0.9972	64.83	0.007	0.022	0.005	0.305
	phytoene	DAD	285	y = 6663.10x + 9.18	0.9991	35.85	0.007	0.022	0.003	0.424
	phytofluene	DAD	350	y = 4323.63x + 3.57	0.9990	24.47	0.007	0.022	0.004	0.521
	violaxanthin	DAD	450	y = 5815.38x − 8.11	0.9993	17.07	0.003	0.011	0.005	0.298
	zeaxanthin	DAD	450	y = 8550.86x − 0.01	0.9999	6.60	0.001	0.002	0.006	0.294
	zeinoxanthin	DAD	450	y = 4693.59x − 88.46	0.9957	91.29	0.023	0.077	0.021	0.727
	ζ-carotene	DAD	410	y = 8713.13x + 1.68	0.9990	34.71	0.004	0.015	0.004	0.322
	echinenone	DAD	450	y = 10,628.83x + 4.15	0.9994	24.45	0.003	0.011	0.002	0.251
Retinol	Retinol	DAD	325	y = 4578.93x + 18.24	0.9992	17.12	0.005	0.016	0.001	0.289
Co-Q10	coenzyme Q10	DAD	285	y = 16,547.06x + 0.27	1.0000	0.63	0.00004	0.0001	0.001	0.066
Tocols	α-tocopherol	DAD	285	y = 931.78x + 0.27	1.0000	2.03	0.003	0.010	0.012	2.343
	β-tocopherol	DAD	285	y = 1245.35x + 4.11	1.0000	3.64	0.005	0.016	0.008	2.190
	δ-tocopherol	DAD	285	y = 1323.25x −2.23	1.0000	6.35	0.008	0.026	0.012	2.748
	γ-tocopherol	DAD	285	y = 1273.25x + 1.85	1.0000	2.90	0.004	0.012	0.012	2.352
	α-tocopherol	FLD	*	y = 4706.47x + 2.88	0.999	1.20	0.001	0.002	0.001	0.017
	β-tocopherol	FLD	*	y = 12,027.37x + 0.29	1.000	1.29	0.0002	0.001	0.001	0.017
	δ-tocopherol	FLD	*	y = 13,331.08x + 2.19	1.000	0.95	0.0001	0.0004	0.000	0.014
	γ-tocopherol	FLD	*	y = 13,424.93x + 2.72	1.000	0.72	0.0001	0.0003	0.001	0.018
	α-tocotrienol	DAD	285	y = 325.28x − 0.01	0.9993	15.44	0.054	0.181	0.026	6.901
	β-tocotrienol	DAD	285	y = 302.61x − 3.80	0.9996	10.61	0.040	0.133	0.036	4.738
	δ-tocotrienol	DAD	285	y = 288.33x − 4.97	0.9996	5.74	0.025	0.084	0.038	2.430
	γ-tocotrienol	DAD	285	y = 333.40x − 2.05	0.9999	2.81	0.011	0.036	0.032	2.459
	α-tocotrienol	FLD	*	y = 1686.96x − 0.57	0.997	1.72	0.001	0.005	0.003	0.057
	β-tocotrienol	FLD	*	y = 2108.66x + 0.99	0.998	0.88	0.001	0.003	0.002	0.024
	δ-tocotrienol	FLD	*	y = 2460.70x − 0.38	0.999	0.77	0.001	0.002	0.003	0.024
	γ-tocotrienol	FLD	*	y = 2858.81x + 0.14	0.999	0.86	0.001	0.002	0.002	0.025

Values are expressed as means ± standard deviation; SD_RES_: Standard deviation of the residuals; LOD: limit of detection; LOQ: limit of quantification; * 295 nm excitation and 325 nm emission.

**Table 3:** Summary of accuracy data.

Compounds	Detector	Concentration	Quantity Added (μg)	Recorded Amount (μg)	Recovery (%) **	SD (%) **
α-tocopherol	DAD (285 nm)	High	8.035 ± 0.136	7.297 ± 0.208	90.8	2.6
	DAD (285 nm)	Medium	0.160 ± 0.001	0.145 ± 0.005	90.5	3.1
	FLD (*)	Low	0.047 ± 0.001	0.043 ± 0.002	91.5	2.9
Coenzyme Q10	DAD (285 nm)	High	0.619 ± 0.007	0.240 ± 0.005	38.8	0.8
	DAD (285 nm)	Low	0.014 ± 0.001	0.005 ± 0.001	38.4	2.0
Retinol	DAD (325 nm)	High	41.136 ± 0.136	37.337 ± 0.344	90.7	0.8
	DAD (325 nm)	Low	2.947 ± 0.020	2.775 ± 0.067	94.2	2.3
Echinenone	DAD (450 nm)	High	3.132 ± 0.174	2.777 ± 0.070	88.7	2.2
	DAD (450 nm)	Low	0.011 ± 0.001	0.009 ± 0.001	84.6	2.3

* FLD (295 nm excitation and 325 nm emission) ** *n* = 3 replicates.

**Table 4:** Summary of results about repeatability (intra-day assay) and reproducibility (inter-day assay) for the proposed method.

Compounds	Wavelength	Analyte	Concentration	Repeatability	Reproducibility
			Mean ± SD	RSD%	RSD%
	(nm)		(μg/g FW)	(*n* = 3)	(*n* = 9)
Carotenoids	285	phytoene *Z* isomer 1	0.102 ± 0.004	2.87	4.34
		phytoene *Z* isomer 2	0.021 ± 0.002	6.49	9.15
		phytoene *Z* isomer 3	0.020 ± 0.002	3.78	9.97
		15*Z*-phytoene	0.206 ± 0.016	5.16	7.67
		all-*E*-phytoene	0.039 ± 0.002	4.78	4.49
	Σphytoene		0.387 ± 0.025	4.47	6.51
	350	phytofluene isomer 1	0.055 ± 0.005	3.43	8.24
		phytofluene isomer 2	0.095 ± 0.007	7.19	7.81
		phytofluene isomer 3	0.223 ± 0.013	3.49	6.03
		phytofluene isomer 4	0.093 ± 0.005	4.74	5.43
	Σphytofluene		0.466 ± 0.030	4.36	6.41
	410	ζ-carotene isomer 1	0.013 ± 0.001	6.59	9.73
		ζ-carotene isomer 2	0.005 ± 0.0001	4.80	4.54
		ζ-carotene isomer 3	0.025 ± 0.002	5.61	7.36
		ζ-carotene isomer 4	0.046 ± 0.003	4.94	6.54
		ζ-carotene isomer 5	0.030 ± 0.001	2.08	3.79
	Σζ-carotene		0.134 ± 0.010	4.45	6.15
	450	all-*E*-lutein	0.088 ± 0.006	10.19	7.23
		all-*E*-zeaxanthin	0.025 ± 0.002	8.82	7.67
		all-*E*-capsanthin	0.043 ± 0.003	5.75	7.84
		all-*E*-zeinoxanthin	0.037 ± 0.001	2.24	4.07
		all-*E*-β-cryptoxanthin	0.013 ± 0.001	6.52	9.53
		all-*E*-α-carotene	0.012 ± 0.001	6.84	9.13
		9*Z-*α-carotene	0.009 ± 0.0001	2.63	4.83
		all-*E*-β-carotene	0.030 ± 0.002	4.51	5.86
		9*Z-*β-carotene	0.039 ± 0.001	4.47	3.81
	472	lycopene Z isomer	0.012 ± 0.001	3.88	7.13
		all-*E*-lycopene	0.021 ± 0.002	4.62	7.94
	Σlycopene		0.032 ± 0.002	4.33	7.62
	Total carotenoids		1.135 ± 0.079	4.87	6.04
Tocopherols	285	α-tocopherol	1.268 ± 0.121	9.33	9.51
		β-tocopherol	0.106 ± 0.007	6.28	6.64
	Σ tocopherols		1.374 ± 0.127	9.08	9.25
Coenzyme Q10	285	Co-Q10	0.115 ± 0.020	10.21	17.54
		Co-Q10-D *	0.087 ± 0.014	6.19	16.73
	Σ coenzyme Q10		0.202 ± 0.034	8.25	16.86
Retinol	325	retinol	6.862 ± 0.563	6.83	8.20

* Co-Q10-D: coenzyme Q10 derivate.

**Table 5:** Carotenoid, tocopherols, coenzyme Q10 and retinol concentration in human and animal tissue samples (µg compound/g tissue).

	Tissue	Duodenum	Intestine	Pancreas	Spleen	Liver	Adipose Tissue + Muscle	Intestine + Muscle	Liver	Liver
	Matrix	Human	Human	Human	Human	Human	Human	Human	Cow	Chicken
Tocopherols	α-tocopherol	0.065	3.908	0.483	0.458	0.470	1.384	0.413	4.121	3.991
Coenzyme Q10	Co-Q10	0.003	0.023	0.006	nd	0.112	0.004	0.052	nd	0.291
	Co-Q10	0.002	0.011	0.003	nd	0.011	0.002	0.029	nd	0.023
Carotenoids	phytoene *Z* isomer 1	nd	0.005	0.001	nd	nd	0.005	0.008	0.045	0.033
	phytoene *Z* isomer 3	nd	nd	nd	nd	nd	nd	0.004	nd	nd
	15*Z*-phytoene	nd	0.003	nd	nd	0.089	nd	0.025	nd	nd
	all-*E*-phytoene	nd	nd	nd	nd	nd	nd	0.011	nd	nd
	phytofluene isomer 1	nd	nd	nd	0.009	nd	nd	0.011	nd	nd
	phytofluene isomer 2	nd	0.004	nd	0.009	0.072	nd	0.023	nd	nd
	phytofluene isomer 3	nd	0.003	nd	0.002	nd	nd	0.019	nd	nd
	phytofluene isomer 4	0.003	0.004	nd	0.028	0.113	0.002	0.169	nd	nd
	ζ-carotene isomer1	0.001	0.003	nd	0.007	0.037	0.001	0.010	nd	nd
	ζ-carotene isomer 2	0.004	0.007	nd	0.015	0.069	0.002	0.029	nd	nd
	ζ-carotene isomer 3	0.002	0.006	nd	0.007	0.040	0.001	0.020	nd	nd
	13*Z*-lutein	0.004	nd	nd	0.001	nd	0.001	0.004	0.028	0.018
	di-*Z*-zeaxanthin	0.002	nd	nd	nd	nd	nd	nd	0.046	0.043
	all-*E*-lutein	0.055	0.005	0.001	0.047	0.031	0.008	0.037	0.387	0.296
	all-*E*-zeaxanthin	0.010	nd	nd	0.018	0.044	0.006	0.026	0.088	0.074
	all-*E*-capsanthin	0.002	0.026	0.004	nd	0.021	0.029	0.027	nd	nd
	all-*E*-zeinoxanthin	nd	0.005	nd	0.033	0.063	0.005	0.046	0.076	0.049
	9*Z*-zeaxanthin	nd	nd	nd	nd	nd	nd	nd	0.008	0.006
	all-*E*-β-cryptoxanthin	0.003	0.007	0.001	0.055	0.096	0.009	0.095	nd	nd
	15*Z*-β-carotene	nd	nd	nd	nd	0.006	nd	0.001	nd	nd
	13*Z*-β-carotene	0.001	nd	0.001	0.011	0.070	0.001	0.017	nd	nd
	all-*E*-α-carotene	0.002	0.003	nd	0.015	0.053	0.003	0.011	0.004	0.008
	9*Z*-α-carotene	nd	0.001	nd	0.004	0.012	nd	0.002	nd	0.003
	All-*E*-β-carotene	0.006	0.009	0.003	0.033	0.342	0.006	0.092	0.004	0.004
	9*Z*-β-carotene	0.002	0.002	0.001	0.006	0.123	0.001	0.017	nd	0.002
	15*Z*-lycopene	nd	nd	nd	0.003	0.031	nd	0.008	nd	nd
	13*Z*-lycopene	0.002	0.003	0.001	0.011	0.170	0.001	0.056	nd	nd
	lycopene *Z* isomer 1	0.002	0.003	0.001	0.008	0.116	0.002	0.029	nd	nd
	lycopene *Z* isomer 2	0.002	0.003	0.001	0.012	0.065	0.004	0.024	nd	nd
	all-*E*-lycopene	0.017	0.014	0.010	0.084	2.307	0.027	0.296	nd	nd
	5*Z*-lycopene	0.005	0.003	0.004	0.044	0.083	0.008	0.034	nd	nd
Retinol	Retinol	nd	nd	0.014	0.094	1.521	0.020	0.054	86.166	79.218
	Retinol-D 1	nd	nd	nd	nd	0.214	nd	nd	0.135	0.211
	Retinol-D 2	nd	nd	nd	nd	4.332	nd	nd	0.315	0.453
	Retinol-D 3	nd	0.007	nd	nd	0.135	nd	nd	0.094	0.203

Retinol-D: retinol derivative.

## Data Availability

The original contributions presented in this study are included in the article. Further inquiries can be directed to the corresponding authors.

## Abbreviations

The following abbreviations are used in this manuscript:

Co-Q10	Coenzyme Q_10_
RRLC	Rapid Resolution Liquid Chromatography
DAD	Diode Array Detector
FLD	Fluorescence Detector
LOD	Limit of Detection
LOQ	Limit of Quantification
BHT	Butylated Hydroxytoluene
RSD	Relative Standard Deviation

## References

[1] Meléndez-Martínez A.J., Böhm V., Borge G.I., Cano M.P., Fikselová M., Gruskiene R., Lavelli V., Loizzo M.R., Mandić A., Mapelli-Brahm P. (2021). Carotenoids: Considerations for Their Use in Functional Foods, Nutraceuticals, Nutricosmetics, Supplements, Botanicals and Novel Foods in the Context of Sustainability, Circular Economy and Climate Change. Annu. Rev. Food Sci. Technol..

[2] Meléndez-Martínez A.J. (2019). An Overview of Carotenoids, Apocarotenoids, and Vitamin A in Agro-Food, Nutrition, Health, and Disease. Mol. Nutr. Food Res..

[3] Kong R., Cui Y., Fisher G.J., Wang X., Chen Y., Schneider L.M., Majmudar G. (2016). A Comparative Study of the Effects of Retinol and Retinoic Acid on Histological, Molecular, and Clinical Properties of Human Skin. J. Cosmet. Dermatol..

[4] Beer K., Singh A., Ravi S.C., Gupta A.K., Kumar A., Sharma M.M. (2024). A Comprehensive Review on the Role of Vitamin A on Human Health and Nutrition. J. Environ. Biol..

[5] D’Ambrosio D.N., Clugston R.D., Blaner W.S. (2011). Vitamin A Metabolism: An Update. Nutrients.

[6] Nesaretnam K. (2008). Multitargeted Therapy of Cancer by Tocotrienols. Cancer Lett..

[7] Latib F., Zafendi M.A.I., Mohd Lazaldin M.A. (2024). The Use of Vitamin E in Ocular Health: Bridging Omics Approaches with Tocopherol and Tocotrienol in the Management of Glaucoma. Food Chem. Mol. Sci..

[8] Traber M.G., Atkinson J. (2007). Vitamin E, Antioxidant and Nothing More. Free Radic. Biol. Med..

[9] Wagner K.H., Kamal-Eldin A., Elmadfa I. (2004). Gamma-Tocopherol—An Underestimated Vitamin?. Ann. Nutr. Metab..

[10] Hernández-Camacho J.D., Bernier M., López-Lluch G., Navas P. (2018). Coenzyme Q10 Supplementation in Aging and Disease. Front. Physiol..

[11] Gutierrez-Mariscal F.M., Yubero-Serrano E.M., Villalba J.M., Lopez-Miranda J. (2019). Coenzyme Q10: From Bench to Clinic in Aging Diseases, a Translational Review. Crit. Rev. Food Sci. Nutr..

[12] Engelmann N.J., Clinton S.K., Erdman J.W. (2011). Nutritional Aspects of Phytoene and Phytofluene, Carotenoid Precursors to Lycopene. Adv. Nutr..

[13] Meléndez-Martínez A.J., Vicario I.M., Heredia F.J. (2015). Estabilidad de Los Pigmentos Carotenoides En Los Alimentos. Arch. Latinoam. Nutr..

[14] Khachik F., Carvalho L., Bernstein P.S., Muir G.J., Zhao D.Y., Katz N.B. (2002). Chemistry, Distribution, and Metabolism of Tomato Carotenoids and Their Impact on Human Health. Exp. Biol. Med..

[15] Schmitz H.H., Poor C.L., Wellman R.B., Erdman J.W. (1991). Concentrations of Selected Carotenoids and Vitamin A in Human Liver, Kidney and Lung Tissue. J. Nutr..

[16] Stahl W., Schwarz W., Sundquist A.R., Sies H. (1992). Cis-Trans Isomers of Lycopene and Β-Carotene in Human Serum and Tissues. Arch. Biochem. Biophys..

[17] Peng Y.M., Peng Y.S., Lin Y. (1993). A Nonsaponification Method for the Determination of Carotenoids, Retinoids, and Tocopherols in Solid Human Tissues. Cancer Epidemiol. Biomark. Prev..

[18] Clinton K., Emenhiser C., Schwartz J., Bostwick G., Williams W., Moore B.J., Erdman J.W. (1996). Lycopene in the and Retinol. Cancer Epidemiol. Biomark. Prev..

[19] Gamboa-Pinto A.J., Rock C.L., Ferruzzi M.G., Schowinsky A.B., Schwartz S.J. (1998). Cervical Tissue and Plasma Concentrations of α-Carotene and β-Carotene in Women Are Correlated. J. Nutr..

[20] Yeum K.J., Ahn S.H., De Paiva S.A.R., Lee-Kirn Y.C., Krinsky N.I., Russell R.M. (1998). Correlation between Carotenoid Concentrations in Serum and Normal Breast Adipose Tissue of Women with Benign Breast Tumor or Breast Cancer. J. Nutr..

[21] El-Sohemy A., Baylin A., Kabagambe E., Ascherio A., Spiegelman D., Campos H. (2002). Individual Carotenoid Concentrations in Adipose Tissue and Plasma as Biomarkers of Dietary Intake. Am. J. Clin. Nutr..

[22] Craft N.E., Haitema T.B., Garnett K.M., Fitch K.A. (2004). Carotenoid, Tocopherol, and Retinol Concentrations in Elderly Human Brain. J. Nutr. Health Aging.

[23] Qin J., Yeum K.J., Johnson E.J., Krinsky N.I., Russell R.M., Tang G. (2008). Determination of 9-Cis β-Carotene and ζ-Carotene in Biological Samples. J. Nutr. Biochem..

[24] Chung H.Y., Ferreira A.L.A., Epstein S., Paiva S.A.R., Castaneda-Sceppa C., Johnson E.J. (2009). Site-Specific Concentrations of Carotenoids in Adipose Tissue: Relations with Dietary and Serum Carotenoid Concentrations in Healthy Adults. Am. J. Clin. Nutr..

[25] Vishwanathan R., Neuringer M., Snodderly D.M., Schalch W., Johnson E.J. (2013). Macular lutein and zeaxanthin are related to brain lutein and zeaxanthin in primates. Nutr. Neurosci..

[26] Zhong G., Kirkwood J., Won K.J., Tjota N., Jeong H., Isoherranen N. (2019). Characterization of Vitamin A Metabolome in Human Livers with and without Nonalcoholic Fatty Liver Disease. J. Pharmacol. Exp. Ther..

[27] Harari A., Coster A.C.F., Jenkins A., Xu A., Greenfield J.R., Harats D., Shaish A., Samocha-Bonet D. (2020). Obesity and Insulin Resistance Are Inversely Associated with Serum and Adipose Tissue Carotenoid Concentrations in Adults. J. Nutr..

[28] Montero R., Sánchez-Alcázar J.A., Briones P., Hernández Á.R., Cordero M.D., Trevisson E., Salviati L., Pineda M., García-Cazorla A., Navas P. (2008). Analysis of Coenzyme Q10 in Muscle and Fibroblasts for the Diagnosis of CoQ10 Deficiency Syndromes. Clin. Biochem..

[29] Kane M.A., Folias A.E., Napoli J.L. (2009). HPLC/UV Quantitation of Retinal, Retinol, and Retinyl Esters in Serum and Tissues. Anal. Biochem..

[30] Uner B., Celik A., Ergin A.D., Altay Benetti A., Benetti C. (2024). Enhancement of In-Vivo Cellular Uptake of Coenzyme Q10 Using Saponin Derivatives in RTALAP Transgenic Mice Model. J. Drug Deliv. Sci. Technol..

[31] Arnold S.L.M., Amory J.K., Walsh T.J., Isoherranen N. (2012). A Sensitive and Specific Method for Measurement of Multiple Retinoids in Human Serum with UHPLC-MS/MS. J. Lipid Res..

[32] Ruiz-Jiménez J., Priego-Capote F., Mata-Granados J.M., Quesada J.M., Luque de Castro M.D. (2007). Determination of the Ubiquinol-10 and Ubiquinone-10 (Coenzyme Q10) in Human Serum by Liquid Chromatography Tandem Mass Spectrometry to Evaluate the Oxidative Stress. J. Chromatogr. A.

[33] Stinco C.M., Benítez-González A.M., Meléndez-Martínez A.J., Hernanz D., Vicario I.M. (2019). Simultaneous Determination of Dietary Isoprenoids (Carotenoids, Chlorophylls and Tocopherols) in Human Faeces by Rapid Resolution Liquid Chromatography. J. Chromatogr. A.

[34] Gleize B., Steib M., André M., Reboul E. (2012). Simple and Fast HPLC Method for Simultaneous Determination of Retinol, Tocopherols, Coenzyme Q10 and Carotenoids in Complex Samples. Food Chem..

[35] Melendez-Martinez A.J., Stinco C.M., Liu C., Wang X.D. (2013). A Simple HPLC Method for the Comprehensive Analysis of Cis/Trans (Z/E) Geometrical Isomers of Carotenoids for Nutritional Studies. Food Chem..

[36] Honda M. (2021). Carotenoid Isomers: A Systematic Review of the Analysis, Biological Activity, Physicochemical Property, and Methods for Isomerization.

[37] (1999). Exactitud (Veracidad y Precisión) de Resultados y Métodos de Medición. Parte 2: Método Básico Para La Determinación de La Repetibilidad y La Reproducibilidad de Un Método de Medición Normalizado.

[38] Miller J., Miller J.C. (2010). Statistics and Chemometrics for Analytical Chemistry.

[39] Britton G., Liaaen-Jensen S., Pfander H. (1994). Carotenoids, Volume 1B: Spectroscopy.

[40] Stinco C.M., Benítez-González A.M., Hernanz D., Vicario I.M., Meléndez-Martínez A.J. (2014). Development and Validation of a Rapid Resolution Liquid Chromatography Method for the Screening of Dietary Plant Isoprenoids: Carotenoids, Tocopherols and Chlorophylls. J. Chromatogr. A.

[41] Turkowicz M.J., Karpińska J. (2013). Analytical Problems with the Determination of Coenzyme Q10 in Biological Samples. BioFactors.

[42] Mosca F., Fattorini D., Bompadre S., Littarru G.P. (2002). Assay of Coenzyme Q10 in Plasma by a Single Dilution Step. Anal. Biochem..

[43] Abdul-Rasheed O.F., Farid Y.Y. (2009). Development of a New High Performance Liquid Chromatography Method for Measurement of Coenzyme Q10 in Healthy Blood Plasma. Saudi Med. J..

[44] Boulet L., Alex B., Clavey N., Martinez J., Ducros V. (2020). Simultaneous Analysis of Retinol, Six Carotenoids, Two Tocopherols, and Coenzyme Q10 from Human Plasma by HPLC. J. Chromatogr. B Anal. Technol. Biomed. Life Sci..

[45] Antwi S.O., Steck S.E., Su L.J., Hebert J.R., Zhang H., Craft N.E., Fontham E.T.H., Smith G.J., Bensen J.T., Mohler J.L. (2016). Carotenoid Intake and Adipose Tissue Carotenoid Levels in Relation to Prostate Cancer Aggressiveness among African-American and European-American Men in the North Carolina–Louisiana Prostate Cancer Project (PCaP). Prostate.

[46] Bhagavan H.N., Chopra R.K. (2006). Coenzyme Q10: Absorption, Tissue Uptake, Metabolism and Pharmacokinetics. Free Radic. Res..

